# Buchu (Agathosma betulina and A. crenulata): Rightfully Forgotten or Underutilized?

**DOI:** 10.3389/fphar.2022.813142

**Published:** 2022-02-07

**Authors:** Thomas Brendler, Mona Abdel-Tawab

**Affiliations:** ^1^ Department of Botany and Plant Biotechnology, University of Johannesburg, Johannesburg, South Africa; ^2^ Plantaphile, Collingswood, NJ, United States; ^3^ Traditional Medicinals Inc., Rohnert Park, CA, United States; ^4^ Central Laboratory of German Pharmacists, Eschborn, Germany; ^5^ Institute of Pharmaceutical Chemistry, Johann Wolfgang Goethe University, Frankfurt am Main, Germany

**Keywords:** buchu, ethnobotany, commercialization, pharmacological activity, phytochemical composition

## Abstract

Today, the term *buchu* refers to the two species in commerce, *Agathosma betulina* (P.J.Bergius) Pillans and *Agathosma crenulata* (L.) Pillans (Rutaceae). Its traditional use in urinary tract infections and related ailments made it a popular remedy, specifically in the US, in 19th century, but with the advent of antibiotics it became largely obsolete. Recent focus is on technological use and on the essential oil for use in the perfume and food-flavouring industry. A review of the scarce pharmacological research revealed moderate antimicrobial activity for a leaf extract but not the essential oil of both species in the MIC assay. In the 5-lipoxygenase (5-LO) assay the essential oil of both species revealed IC_50_ values of 50.37 ± 1.87 μg/ml and 59.15 ± 7.44 μg/ml, respectively. In another study 98% inhibitory activity was determined for 250 μg/ml of an ethanolic extract of *A. betulina* on cyclooxygenase (COX)-1 and a 25% inhibitory activity on COX-2. Analgesic activity of an ethanolic extract of *A. betulina* was shown in mice. Moderate antioxidant activity was determined for methanol:dichlormethane extracts of *A. betulina* and *A. crenulata* and an aqueous extract of *A. betulina* showed a Trolox equivalent antioxidant capacity (TEAC) of 11.8 µM Trolox. Recent *in vitro* studies with a commercial aqueous extract of *buchu* revealed increased uptake of glucose added to 3T3-L1 cell line, significant inhibition of the respiratory burst of neutrophils and monocytes, reduction in the expression of adhesion molecules and inhibition of the release of IL-6 and TNF-α. In diabetic rats the ingestion of aqueous *buchu* extract completely normalized the glucose level and in rats receiving a high fat diet the consumption of aqueous *buchu* extract resulted in less weight gain and less intraperitoneal fat gain as well as reduction of elevated blood pressure to normal associated with cardioprotective effects. Limitations in the hitherto conducted research lie in the undisclosed composition of the *buchu* extracts used and the difficulty in extrapolating data from animal studies to humans. Health claims for *buchu* products need to be substantiated by randomized, double-blind and placebo-controlled studies. Only then can they be promoted for their true therapeutic potential.

## Introduction

*Agathosma* is a genus of 150 species of flowering plants in the family Rutaceae indigenous to South Africa. The two species in commerce are now known as *A. betulina* (P.J.Bergius) Pillans and *A. crenulata* (L.) Pillans*.* Their common name *buchu*, however, was historically applied to multiple aromatic species of this and other genera. Traditionally, *buchu* has been used by the Khoisan for spiritual and medicinal purposes (Smith, 1966). Initially noted by the early settlers, knowledge and use of *buchu* spread to Europe and later to the United States (US). *Buchu* belongs to a handful of Southern African medicinal plants which reached international markets through colonial interests and entrepreneurship, more or less unaffected by barriers of entry which were more recently introduced by health product regulations in the target markets as well as bioprospecting legislation in the countries of origin (Brendler et al., 2008; Brendler, 2009; Stander et al., 2019; Brendler, 2020; Brendler, 2021; Brendler and Cock, 2021; Brendler et al., 2021). It has been compendial since 1826 for its diuretic effects and use in the treatment of genito-urinary tract infections, however, became obsolete in the 20th century due to the sparsity of scientific evidence for its efficacy and the advent of antibiotics. Today’s interest is focused on technological use and the essential oil for use in perfumes and as a flavouring agent. Nevertheless, *buchu* is still found in numerous herbal preparations, sold over-the-counter (OTC) or on the internet, for promoting health and treatment of urinary tract disorders. Despite its unique and exciting history, many questions regarding *buchu’s* pharmacological properties and potential therapeutic effects remain to be answered.

## History of Buchu

### Taxonomy

**FIGURE 1:**
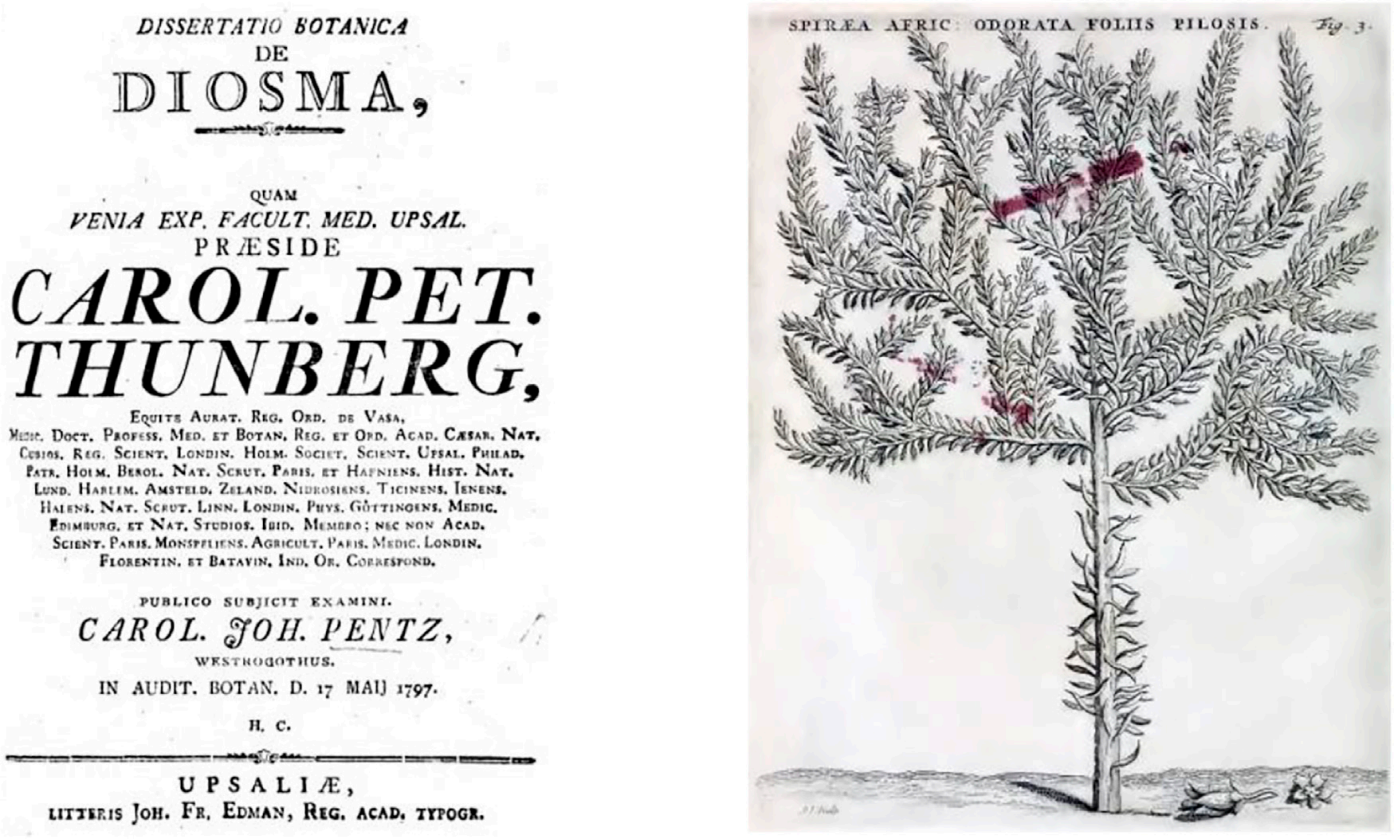
Title page of Thunberg’s Dissertatio Botanica de Diosma (1799), *Spiraea Africana* odorata in Commelin (1706).

Nomenclature of *buchu* is complicated and species identification hampered by historical references omitting authorities. Linné first recorded the genus as *Diosma* in 1756 (Linné, 1756), specifically *D. crenulata* and *D. crenata*, followed by Thunberg in his *Prodromus* (Thunberg, 1794) and his botanical thesis dedicated to *Diosma* (Thunberg, 1797) (Figure 1). Ecklon and Zeyher introduced the genus as *Barosma* and provided detailed botanical and geographical data (Ecklon and Zeyher, 1835). Until the authoritative revision by Pillans, multiple authorities named and renamed species, therefore, it seems appropriate to include here the full synonymy for the two species of *Agathosma* in commerce, while botanical descriptions of the species can be found in the revision (Pillans, 1950):

*A. crenulata* (L.) Pillans = *Diosma crenulata* L.; *D. crenata* L.; *D. latifolia* Andrews; *D. serratifolia* Curt.; *Parapetalifera odorata* Wendl.; *Barapelutiflora serrata* Wendl.; *Barosma odorata* Willd.; *Baryosma odorata*, *B. serratifolia* Roem. & Schultes; *Bucco crenata* Roem. & Schultes; *Adenandra cordata*, *A. serratifolia* Link; *Barosma serratifolia* Willd.; *Diosma odorata* DC.; *Barosma crenata* Sweet; *Agathosma latifolia* Loud.; *Barosma crenulata* Hook.; *B. eckloniana* O. Berg.

*A. betulina* (P.J.Bergius) Pillans = *Hartogia betulina* P.J.Bergius; *Diosma betulina* Thunb.; *Bucco betulina* Roem. & Schultes; *Diosma crenata* Lodd.; *Barosma betulina* Bartl. & Wendl.

Common names include *boegoe, boechoe, boekoe, boggoa, bookoo, bouchou, bugu, buccho, bucchuu, bucco, buchu, bucku and buku* (Smith, 1966).

### Ethnobotany and Ethnobiology

Before moving on to discuss the records of traditional use of *buchu* made by early settlers, colonists, and explorers, it must be stressed that the knowledge of *buchu* and its medicinal properties by the Khoisan precedes written records, probably by centuries. The Digital Bleek and Lloyd^1^, a digital archive of the ethnographical exploration of the Khoisan people, lists the use of *buchu* in multiple every-day, spiritual and medicinal contexts. The Khoisan and other indigenous peoples considered multiple aromatic species as *buchu* and used them in dance rituals, for anointment, beautification, perfume, and also as medicine (Smith, 1966; Low, 2007). Within the realm of those recorded uses, traditional knowledge is owned by the Khoisan and should be attributed and respected as such (Low, 2007).

**FIGURE 2:**
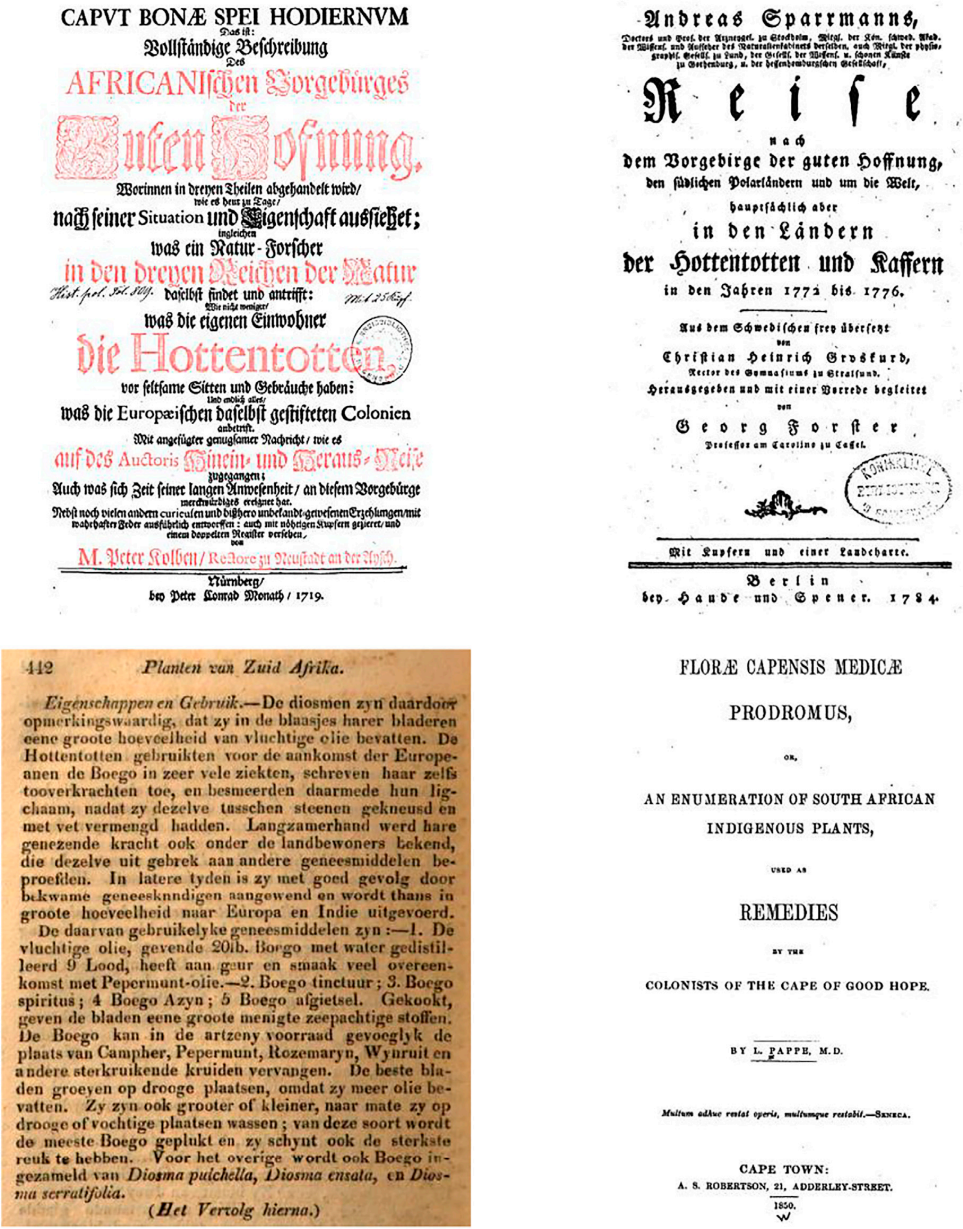
Title page of Kolb (1719), title page of Sparrmann (1784), excerpt from Ecklon (1826), title page of Pappe (1850).

The first published record of *boggoa* leaves being used in tribal dance practices was made by an early settler in 1668 and later reiterated in other settlers’ records (Dapper et al., 1933; Smith, 1966). Interestingly, and contrary to secondary sources, neither van der Stel’s travel journal from 1685 (van der Stel, 1979) nor the *Codex Witsenii* of 1692 (Wilson, 2002) included *buchu* species. Commelin, in 1706, described three species in his *Horti Medici Amstelaedamensis* as *Spiraea Africana* (Commelin, 1706) and noted the great importance to the local “hottentots” (Figure 1). Kolb first mentioned the common name *buchu*, not *boegoe* as claimed by Smith (1966), and described the use of the pleasant smelling, dried and powdered leaves for aches and anointments (Kolb, 1719) (Figure 2). This constitutes the first record for medicinal use, again contrary to Smith, who ascribed the first medicinal use record to Sparrman et al. (1784) (Figure 2), who indeed reported on the use of *bucku* for anointment and as a strong medicine, but without providing further detail. He also alluded to different species being plentiful, while one of them is particularly valued: “*Some species are common in the Cape, one, however, to be found somewhere near the gold river, is so precious that one thimble of the powder is paid for with a lamb*” (author’s transl.) (Sparrman et al., 1784). Burchell, in his *Travels in the interior of southern Africa* recorded names and uses (Burchell, 1822). Latrobe followed shortly after and for the first time noted the use of *buchu* brandy: “*… we found the larger species of bukku, one of the most aromatic, medicinal, plants in the country, and justly esteemed for its healing properties. Its leaves steeped in brandy or vinegar, and the bottle placed in the heat of the sun, emit an unctuous juice, by which the fluid is rendered as thick as honey, and applied particularly for the healing of contusions, sores, and all external complaints. The Hottentots also use it for inward hurts, by mixing a spoonful of it with warm water … ”* (Latrobe, 1818). In the same year, de Candolle praised the pleasant smell of *buchu* essential oil and its spasmolytic properties (De Candolle, 1818).

Between 1826 and 1831, Ecklon published a series of articles in *Het Nederduitsch Zuid-Afrikaansch Tydschrift* on the identity, habitat and virtues of local medicinal plants, and specifically on *boego* (*Diosma crenata*) (Ecklon, 1826). Therein he gave a detailed description and a contemporary summary of uses: *“…Hottentots used the boego in many diseases, even ascribing magical powers to it, and smearing their bodies with it, after they had bruised it between stones and mixed it with fat. … In recent times it has been successfully used by skilled physicians and is now exported in great quantity to Europe and India. … The boego can conveniently replace camphor, peppermint, rosemary, rue, and other strong-smelling herbs in the medical supply.” (author’s transl.)* (Figure 2).

Given that Pappe was associated with Ecklon, and undoubtedly acquainted with Mackrill, Liesching and other contemporaries (see below), his statement that “*I am not aware of any publication on South African Materia Medica, except that of a small dissertation by Thunberg, which appeared in the year 1785*” (Pappe, 1847; Pappe, 1850) seems bizarre; he did, however, add a few details to his description reflecting overseas research, e.g., the identification of diosmin, attribution of medicinal uses to the essential oil, and new uses: as a diuretic, for gout and rheumatism, urinary ailments, as an appetite stimulant, and even cholera (Pappe, 1850) (Figure 2).

Later accounts (Watt and Breyer-Brandwijk, 1962; Smith, 1966) drew from these early sources.

From an ethnobiological point of view *buchu* was a highly prized Khoisan traditional remedy and remained one of the most popular herbal medicines in South-Africa. The traditional use of *buchu* encompasses the treatment of kidney and urinary tract infections, cold, stomach ailments, rheumatism, gout and fever. Externally it was applied as an antiseptic wash to infected wounds and as a compress to relieve swelling, bruising and sprains. In traditional practice *A. betulina* is most commonly taken orally in form of an aqueous infusion, sometimes sweetened with brown sugar, or as tincture in brandy. Other dosage forms include a vinegar infusion for external application as an antiseptic wash or embrocation (South African National Biodiversity Institute, 2005).

### Commercialization

*Buchu* arrived in Europe via two distinct and chronologically almost parallel paths, only one of which is documented in the literature. Generally, Joseph Mackrill (1762–1820) is credited with the introduction of *buchu* into the United Kingdom (UK) around 1815 (Theal, 1908; Gunn and Codd, 1981; Low, 2007), and from there to Ireland and the United States. Since Mackrill had spent time in the US prior to his arrival in the Cape, there may very well also have been a direct (trade) connection, but this could not be confirmed.

Richard Reece (1775–1831), a London pharmacist and wholesaler became the proprietor of *buchu* in the Anglo-Saxon world (Reece, 1822; Reece, 1824; Reece, 1827; Reece, 1836) and on a small scale also into Germany, where Friedrich Jobst, pharmacist in Bavaria became his “broker” around 1825 (Jobst, 1825). But Theal (Theal, 1908) and all citing authors in this singular accreditation underestimate the vibrancy of the Cape colony population and its vast and intricate network into Central Europe: next to the UK primarily to the Netherlands and Germany. Indeed, around the same time, *buchu* was introduced into Central Europe via Amsterdam. A German pharmacist and quinine fabricant in Nordhorn (today in Lower Saxony, Germany), Ernst Firnhaber, who procured chinchona bark from Amsterdam and supplied quinine to the Dutch colonies (Kühle, 1970), in 1826 published a note by Friedrich Ludwig Liesching (1757–1841) on the virtues of *buchu* (Firnhaber, 1826) which also mentioned its already established use in the Netherlands. Firnhaber’s friendship with the editor of the journal, notable scientist, and co-founder of what became the German pharmacists association, Rudolph Brandes, helped spread the word throughout the association, which by 1821 already had over 100 members, and resulted in further publications and scientific investigation (Brandes, 1826; Brandes, 1827).

Mackrill and Liesching were contemporaries in the Cape. The medical community was small, it can safely be assumed that they were acquainted. Mackrill, an Englishman, arrived in the Cape from Maryland (US) around 1806 and was admitted to practice as a surgeon in August 1807 (Gunn and Codd, 1981; Glen and Germishuizen, 2010).

At that point in time, Liesching was already well established. He had landed at the Cape at the end of 1787 as surgeon-major of the 1st battalion of the *Württembergische Kapregiment*, which served the Dutch East India Company as mercenaries. The regiment moved on in 1791, but Liesching stayed behind and established himself among the medical community (Deacon and Van Heyningen, 2004). In 1800, he started *Dr Liesching and Company, Apothecaries and Retail Shop* in partnership with Jean Jacques von Ziegler (1766-?) at 61 Loop Street, Cape Town, which was to become one of the largest apothecary shops in the colony (Burrows, 1958). There they were joined by Carl Ferdinand Heinrich von Ludwig (1784–1847), at the time pharmacist in Amsterdam, who applied for a position as a pharmacy assistant to Liesching in 1805, and was approved as an apothecary by the *Supreme Medical Committee*—a body set up to control practice of medicine and pharmacy—in 1807 (Price, 1974). *Buchu*—the universal panacea—featured prominently in the pharmacy’s portfolio for decades, even after *Liesching & Co.* was sold and renamed *De Engel Apotheek* in 1836 (McMagh, 1992). In 1808, Liesching and von Ziegler established a botanical garden at The Knoll (Figure 2), on Kloof Road, above Botany (now Bantry) Bay. The main building of the estate was demolished in the early 2000s to make room for real-estate development (Hart, 2001). The garden held specimens of useful indigenous and exotic flora (Spohr, 1968; McMagh, 1992). Unfortunately, no exact records of its holdings survive, thus, albeit highly likely, cultivation of *buchu* can only be assumed.

As of 1810 Mackrill practiced at 10 Burg Street, Cape Town, where he was visited by Burchell (1822). By 1814, Mackrill had sold his residence and set up an experimental farm for the cultivation of tobacco in Somerset West (Skead, 2009). During this period, he was collecting (and probably also propagating) specimens of the useful indigenous flora and knowledge about its use (Kannemeyer, 1951; Gunn and Codd, 1981; Glen and Germishuizen, 2010). Purportedly, his notes provided input to Pappe’s *Florae Capensis Medicae Prodromus* (Pappe, 1850).

In 1816, Christian Ignatius Latrobe (1758–1836), clergyman of the Moravian church, visited both Mackrill on his Somerset farm and Liesching at Botany Bay (Latrobe, 1818). Latrobe’s account also mentioned a Reverend Hesse, who was in his company on the visit to Liesching, and, according to Smith (1966), sent (or took) seeds and specimens of *buchu* to Germany. According to Smith, Latrobe and/or Hesse were likely also responsible for *buchu* being sent to Moravian missionaries in Calcutta and Madras to treat intestinal colic.

In 1815, Ludwig married into money to evolve as Baron von Ludwig of the Ludwigsburg Gardens, three acres of land in Kloof Street (now Tamboerskloof), Cape Town, which he started to develop in 1829. The gardens, however, were more focused on naturalizing exotic species. To which extent, if at all, he utilized them for the propagation of native species and whether these were part of his business operation is unclear. One fact stands out, however: throughout the years he was collecting native plants with fellow countryman Ludwig Beil near Swellendam, in the Cape Flats, Somerset East, Worcester, Tulbagh, Karsrivier, and Potberg, and in 1838 both accompanied German scientist C.F.F. Krauss on a collection trip to Natal, affording plenty of opportunity to pass on knowledge about *buchu* (Bradlow, 1965).

**FIGURE 3:**
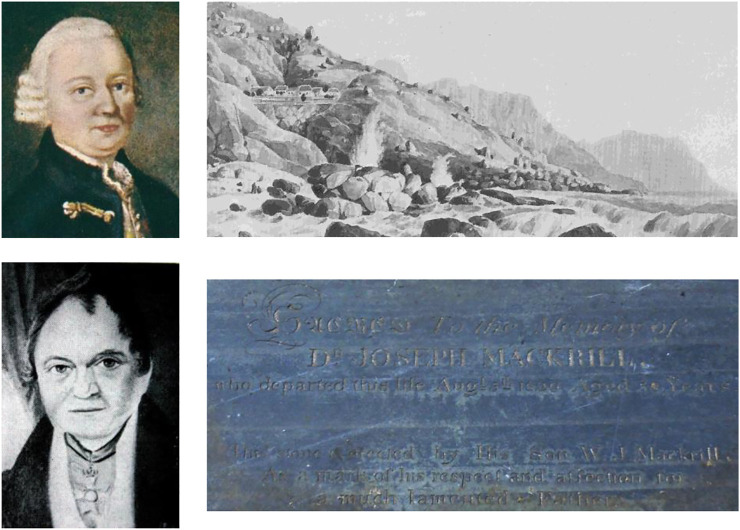
Friedrich Ludwig Liesching, Liesching’s cottages at Botany Bay (1832), Baron von Ludwig (∼1840), Joseph Mackrill’s gravestone at Maitland cemetery, Cape Town.

Thus, not only both Mackrill and Liesching, but also their respective associates (Figure 3) should all be considered to have had a hand in the introduction of *buchu* to Europe and the US.

**FIGURE 4:**
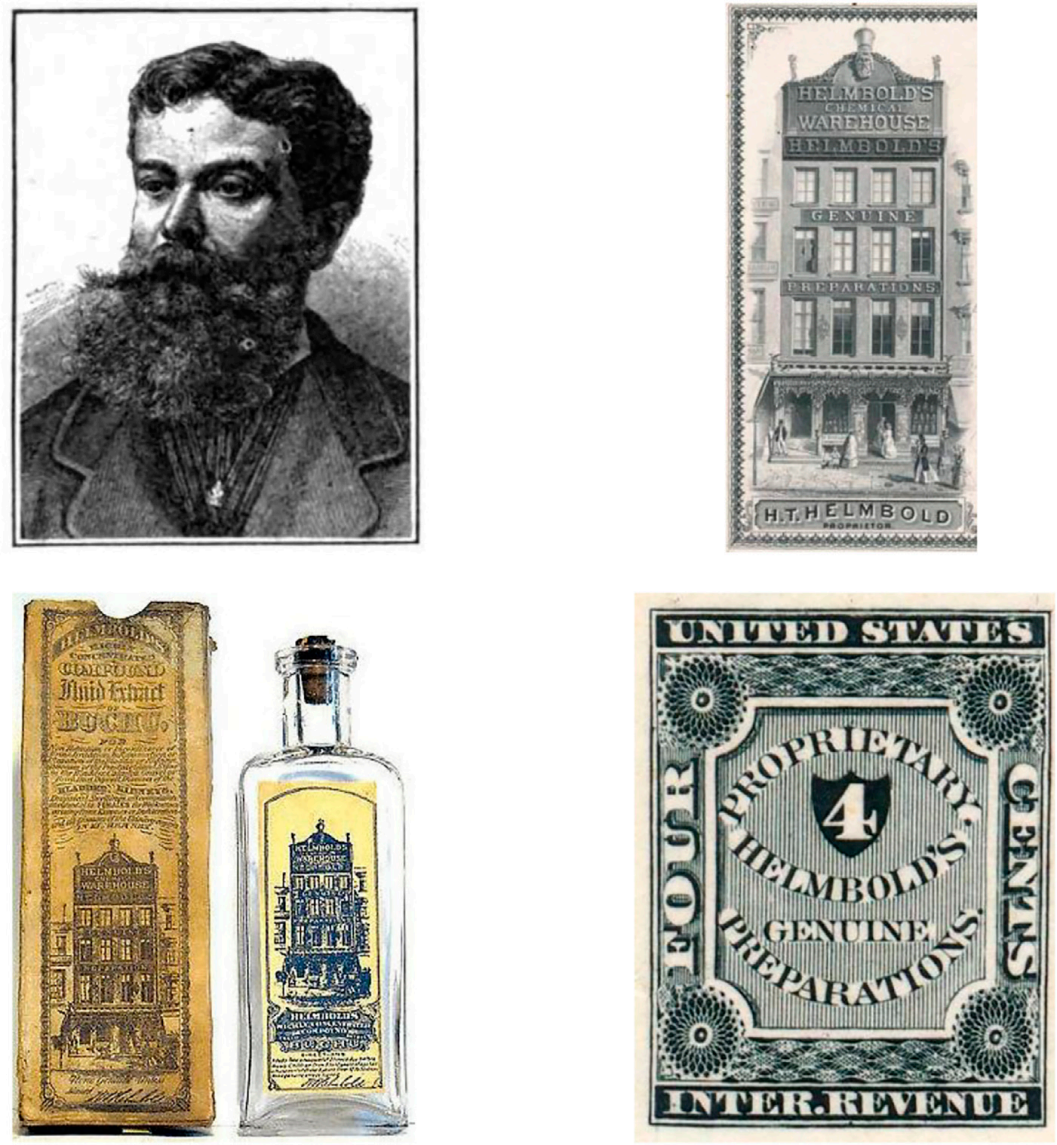
Helmbold ∼1871, one of his storefronts, Helmbold’s extract of *buchu,* his proprietary postage stamp.

*Buchu* products quickly gained popularity, however, less so in Central Europe, but rather in the UK and the US. Unfortunately, there are no epidemiological records for urinary tract ailments in the 19th century for either region. Therefore, no correlations with the popularity of *buchu* can be made. The immense success of *buchu* preparations in the US can thus only be attributed to the marketing practices of manufacturers of patent formulas at the time. One of the most (in)famous protagonists was Henry T. Helmbold (1826–1892), who started his patent medicine business in 1846 as a retail druggist with “*Helmbold’s Extract Buchu—cures diabetes, gravel, brick-dust deposits, irritations of the bladder and diseases arising from exposure or imprudence, etc.*” and other medicines. He opened his first store in Philadelphia in 1850, the largest and best-known in New York in 1862. By 1865 Helmbold’s *buchu* was the bestselling patent medicine on the US market. For this, he spent enormous amounts of money on advertising, mostly in newspapers: ∼US$ 500,000 (about 10 million US$ today) each for the years 1869–71. For the distribution of his products, Helmbold had his own 4c postage stamp (Figure 4) (The Historian, 1912; Young, 1961).

In 1863, Bedford provided some insight in the wholesale value of *buchu*: long-leaved (*B. serratifolia*) and short-leaved (*B. crenulata*) were traded at 80 and 40 cents per pound, respectively (Bedford, 1863). With the Titanic was lost a shipment of eight bales of *buchu* in its cargo (Anonymous, 1912a). For the variety of products in the US market at the beginning of the 20th century, see e.g., the *Drug Department* of the *Druggist Circular* (Anonymous, 1912b).

*Buchu* remained to be a popular remedy way into the 20th century, interest only began to wane with the discovery of antibiotics and synthetic diuretics. Compton (1922), Lawson and Clark (1932) provided some insight into trade volumes of *buchu* during the 1920s (Table 1). Exports had peaked in 1873 at 400,000 pounds, with the bulk going to the US, some via the UK, but also to Europe (mostly UK and Germany). Meanwhile, in the UK, popularity held steady, but around the turn of the century supply was notoriously short. Random checks in *The Chemist and Druggist* confirm steady sales both for the national market and for re-export (Anonymous, 1897; Anonymous, 1908). Only, in the early 1920s, demand appears to decline (Anonymous, 1922).

**TABLE 1:** Exports of *buchu* 1920–1931^a^.

Year	Exports (in pounds)	Value (in US$)
1920	139,149	246,109
1921	124,842	93,309
1922	124,046	76,608
1923	204,297	129,213
1924	152,657	87,310
1925	198,691	79,966
1926	186,589	42,967
1927	139,444	29,359
1928	203,350	39,648
1929	220,669	38,684
1930	157,919	24,879
1931	197,426	26,622

a the inverse trend of volumes and values is noteworthy, however, impacted by currency fluctuations.

Lawson and Clark (1932) also reported on first cultivation attempts, and the profitability thereof. Cultivation, specifically climatic and soil conditions, preparation, seeding, transplanting, culture, harvesting and drying were detailed by Werner (1949), implying a sufficiently steady interest in the crop.

When axenic cultures of *A. betulina* were inoculated with the soil yeast *Cryptococcus laurentii* and cultivated for 5 months under glasshouse conditions, the root growth increased by 51% (Cloete et al., 2009).

### Scientific Investigation and Compendial Representation

Over the 100 years following its introduction to Europe and the US, *buchu* became the subject of multiple scientific investigations and publications. Reece first published on *buchu* in the *Monthly Gazette of Health* (his own “journal”) in 1822 (Reece, 1822). Case reports by McDowell on the efficacy of *buchu* leaf infusions and tinctures in urinary ailments followed (McDowell, 1824), laying the foundation for the inclusion in the Dublin pharmacopoeia (Anonymous, 1826). Another account by Reece followed in 1824 (Reece, 1824) and in 1825, Jackson reported on the use of *buchu* from Calcutta (see above) (Jackson, 1825).

Following the introduction to Germany (Jobst, 1825; Brandes, 1826; Firnhaber, 1826), Jorritsma reported from Amsterdam, on 1) the connection of Liesching to a wholesale pharmacy in Amsterdam (Gebroeders Rouffaer) and 2) several case reports on the successful treatment of urinary tract ailments in the Netherlands (Jorritsma, 1826). *Richard’s Medizinische Botanik* (Kunze, 1826) contained a detailed botanical description of *fol. Diosmae crenatae*, followed by a reiteration of all previously mentioned sources. Noteworthy is a footnote to the account that mentioned a supplier of *buchu* leaves, Brückner, Lampe & Co., a wholesale pharmacy founded in 1750 in Merseburg (Germany), at the time headquartered in Leipzig with subsidiaries in Berlin and Hamburg and business relations reaching as far as Russia and the Unites States (Dufour von Féronce, 1900). The fact that they had 40 pounds of *buchu* readily available suggests a lively trade in Germany at the time.

**FIGURE 5:**
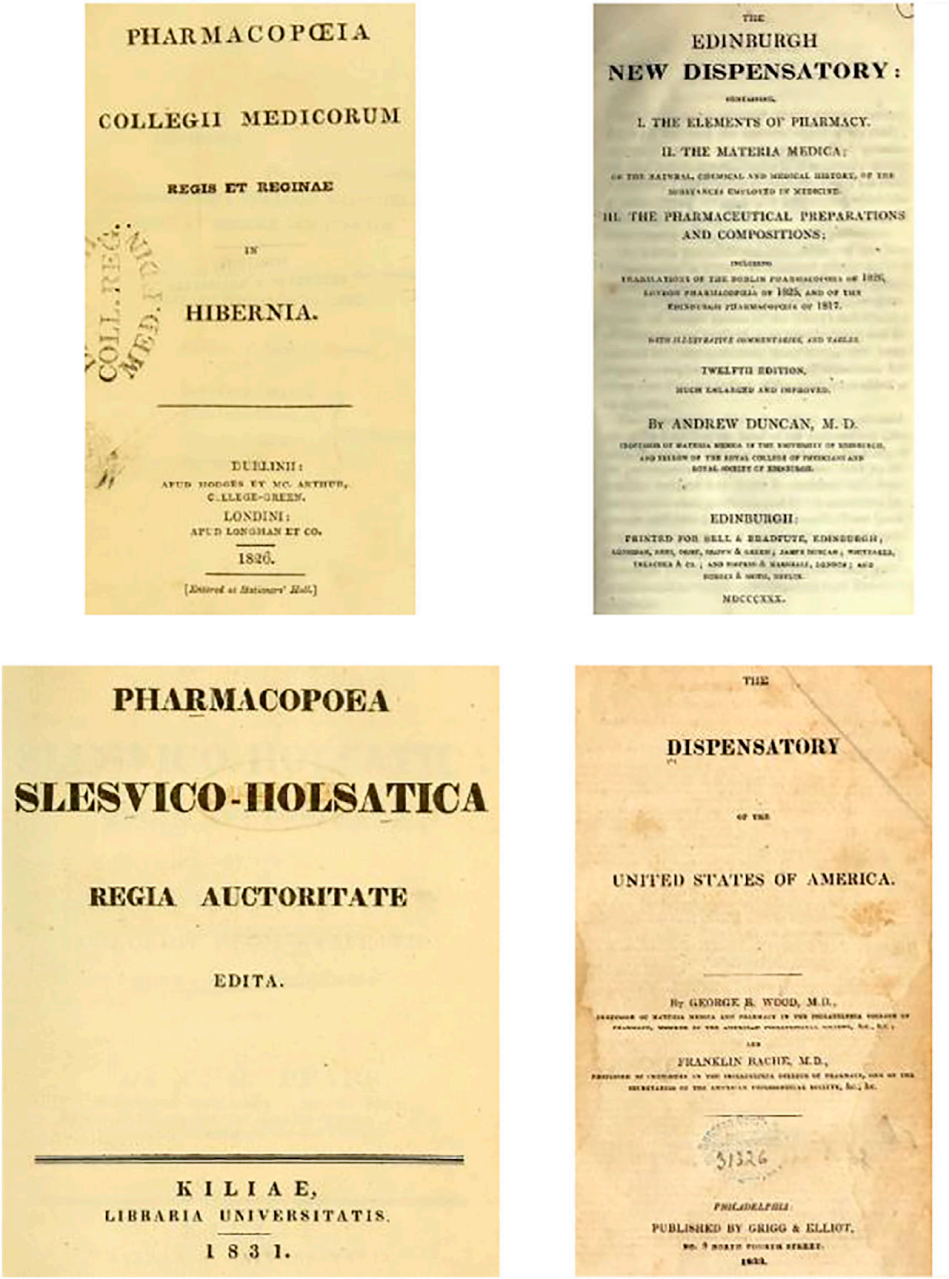
Title pages of the earliest pharmacopeia entries for *buchu* in chronological order: Dublin pharmacopoeia 1826, Edinburgh dispensatory 1830, Schleswig-Holstein pharmacopoeia 1831, US dispensatory 1833.

**FIGURE 6:**
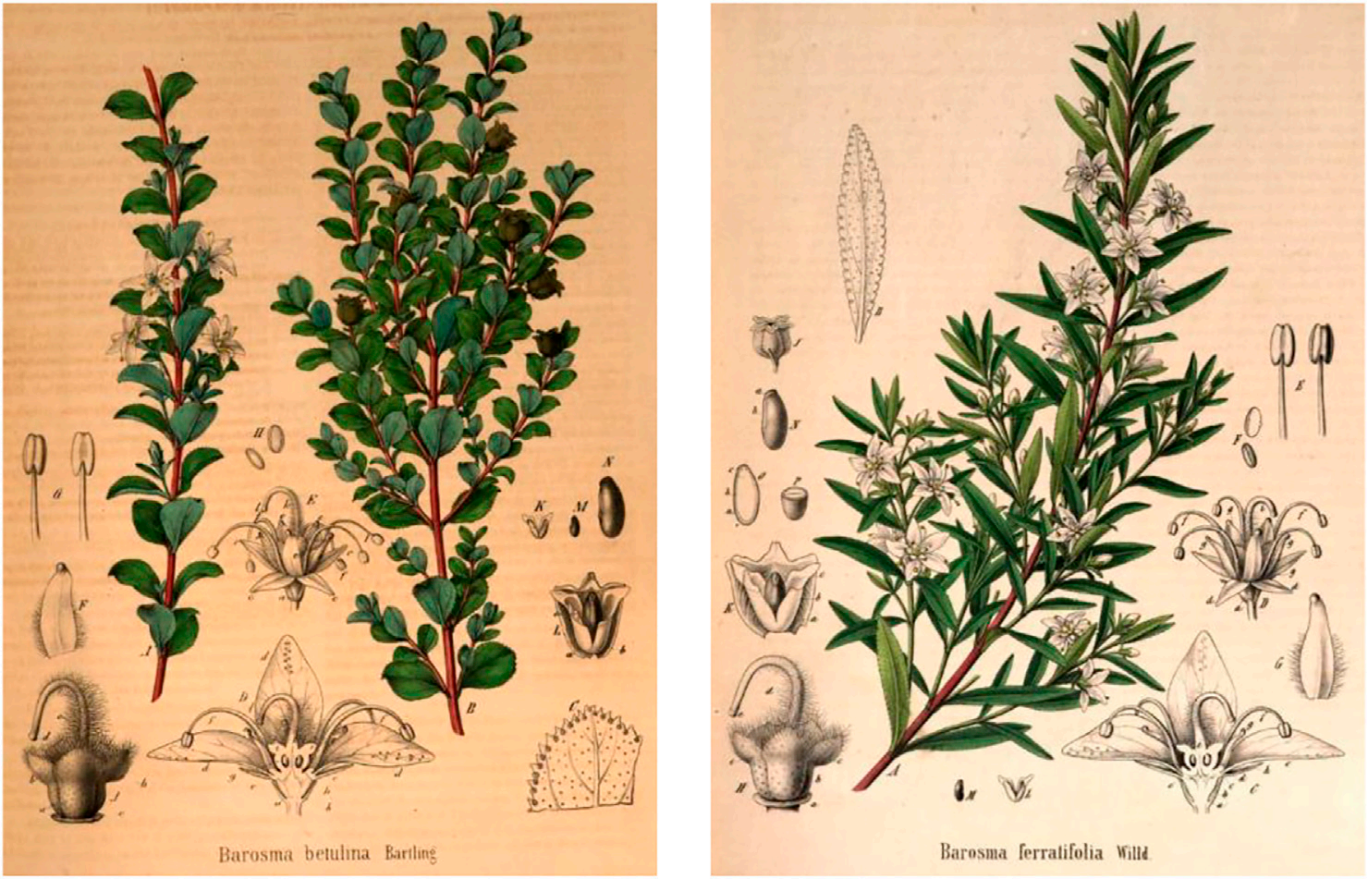
*Agathosma* spp. compendial in the *Pharmacopoeia Borussica* (1846ff) (Berg and Schmidt, 1858).

Cadet de Gassicourt (1827) and Brandes (1827) conducted the first investigations into the chemical composition of *buchu* leaves. Brandes first isolated a substance which he called diosmin. Nees von Esenbeck questioned identity and synonymy of *D. crenata* and *D. serratifolia*, an early indication for admixture and adulteration (Nees von Esenbeck, 1827). Nourij, in his 1827 dissertation, apparently unaware of Brandes’ experiments, reiterated the already known history and proceeded to report his own investigation of various *buchu* preparations confirming the results of Cadet de Gassicourt. He also added a number of case reports to those already presented by Jorritsma (Nourij, 1827). Meanwhile, Reece developed a portfolio of preparations “*as a remedy for morbid irritability of the bladder, prostate gland, spasmodic stricture, irritative gleet, fluor albus, and morbid irritation of the rectum, &c.*” which were advertised in his 1827 *Catalogue Of Drugs* (Reece, 1827). In 1828, Nees von Esenbeck included *D. crenata* and *D. serratifolia* in his *Plantae Officinalis* (Nees von Esenbeck, 1828). Autenrieth’s and Möckel’s dissertations of 1830 were the first treaties with focus on medical applications (Autenrieth, 1830; Möckel, 1830). Long (1831) recommended the use of *buchu* over an extract of belladonna in cases of urethral strictures. By 1836, Reece’s *Popular Catalogue of Drugs* was littered with formulas containing *buchu* and its essential oil (Reece, 1836). Another dissertation (Bruinsma, 1838) summarized advancements in research hitherto and promoted the use of *buchu* as a diuretic, diaphoretic, and stimulant. Two publications of 1847 and 1848 suggested *buchu* for oedema (anasarca, dropsy) (Anonymous, 1847; Hoskins, 1848). Meanwhile, *buchu* had become largely established in European pharmacopoeias (Figures 5, 6, Table 2).

**TABLE 2:** History of compendial representation of *buchu*, compiled from (Anonymous, 1826; Duncan, 1830; Pfaff, 1831; Wood and Bache, 1833; Anonymous, 1835; Anonymous, 1836; Anonymous, 1837; Royal College of Physicians of Edinburgh, 1839; Anonymous, 1842; Anonymous, 1846; Anonymous, 1860; Anonymous, 1916; Bruntz and Jaloux, 1918).

Country/region	*Barosma betulina* (P.J.Bergius) Bartl. & H.L.Wendl., *Diosma betulina* Thunb	*Barosma crenata* (L.) Sweet., *Diosma crenata* L.	*Barosma serratifolia* (Curtis) Willd., *Barosma crenulata* (L.) Hook., *Diosma crenulata* L.
Belgium		(1)—1854	(1)—1854
Denmark	(6)—1893	(5)—1868	(3)—1840
France	(4)—1884	(3)—1866	(4)—1884
Germany	(DAB Erg.-B. 4)—1916	(6)—1846	(6)—1846
Prussia			
Hesse		(2)—1860	(1)—1835
Schlsw.-Holstein		(1)—1831	
Hamburg		(1)—1835	1852^a^ (2)—1837
Lower Saxony		1852^a^ (2)—1837	
Saxony			
Greece		(1)—1837	
Ireland		(2)—1826	
Dublin			
Netherlands	(2)—1871	(1)—1851	(2)—1871
Norway	(2)—1870	(2)—1870	(2)—1870
Portugal	(3)—1876		(3)—1876
Spain	(6)—1884		(6)—1884
Sweden	(7)—1869	(6)—1846	(7)—1869
United Kingdom	(1)—1864	(1)—1864	(1)—1864
London		1836	
Edinburgh		1830, (11) 1839	
USA	(7)—1882^b^	(2)—1833^b^	(3)—1842^b^

Numbers in parentheses refer to the respective editions.

a noted as relevant but not in Pharmacopoea hannoverana nova (Stromeyer, 1852).

b Dispensatory of the US of America (1833), not in the US Pharmacopoeia (USP) until 1842.

In 1861, Strumpf in his *Allgemeine Pharmakopöe*, condensed all hitherto available data into his entry on Folia Bucco (Strumpf, 1861). Flückiger further investigated the microscopic structure of *buchu* leaves and reported a layer of mucilage on their upper side between the epidermis and the mesophyll [Flückiger 1873, cited in Flückiger and Hanbury (1874)]. This description was part of a detailed monograph, which once again summarized the current knowledge.

Jones, with reference to Bedford’s analyses (Bedford, 1863), provided data on ash and soluble matter—relevant for quality control—for the three compendial species at the time (*B. betulina*, *B. crenulata* and *B. serratifolia*) (Jones, 1879). Maisch (1881) and Spica (1885) continued the investigations into the composition of *buchu* essential oil, the former reported on the stearoptene (diosphenol) from *B. betulina* essential oil, the latter disproved the presence of salicylic acid. Shimoyama repeated Flückiger’s experiment and confirmed his results; he also noted the presence of hesperidin crystals (Shimoyama, 1888), the latter being further investigated in terms of distribution and amount by Zenetti (1895). It is noteworthy that both Shimoyama and Zenetti conducted their investigations at least partially on *Diosma alba* Thunb. [synonym of *Coleonema album* (Thunb.) Bartl. & H.L.Wendl.], a related South African Rutaceae species, and then extrapolated their results to compendial *buchu*. Kondakov and Bachtschiew conducted the first comparative elucidation of the essential oil compounds from different sources. Their two oil samples differed significantly in their diosphenol content, leading to the suspicion that they were not derived from the same species. They further referenced and extended an earlier investigation in their laboratory which found three compounds, rather than the previously reported two, a hydrocarbon compound of limonene and dipentene, a ketone (menthone) and diosphenol (Kondakow and Bachtschiew, 1901). McKenzie, (1906) focused on the elucidation of disophenol, or *bucco* camphor, and described its first synthesis via oxidation of oxymethylenmenthone with ozone. Wander revisited the hesperidin in *buchu*, confirmed diosmin, barosmin and hesperidin to be identical, and summarized all there was known on the presence of hesperidin in Rutaceae (Wander, 1925). In 1944, Feldman and Youngken published a pharmacognostic review of *buchu* with many sources cited therein that are not included here (Feldman and Youngken, 1944). This account constituted the most detailed summary of all aspects of identity and quality control at the time.

*Buchu* was monographed in the first edition of the *Dispensatory of the US of America* (Wood and Bache, 1833)—referring to the Dublin pharmacopoeia because the article was not yet listed in the USP. *Buchu* entered the *Primary List of Materia Medica* in the second decennial revision of the USP (Anonymous, 1842), listed as: “*Diosma*. *Buchu*. The leaves of *Diosma crenata*.” A monograph for one preparation was included, *Infusum Diosmae* USP. In the fifth decennial revision (1870), the name of the article changed from *Diosma* to “*Buchu*. The leaves of *Barosma crenata*, and of other species of *Barosma*.” Preparations included *Extractum Buchu Fluidum* and *Infusum Buchu*. *Infusum Buchu* was dismissed from the sixth decennial revision (1880). *Elixir Buchu*, *Elixir Buchu Compositum*, *Elixir Buchu et Potassii Acetatis*, and *Extractum Buchu Fluidum Compositum* appeared in the first edition of the *National Formulary* (NF) (American Pharmaceutical Association, 1888). Squire summarized compendial monographs from British, US and other pharmacopoeias and noted for the *British Pharmacopoeia* (BP) 1898 an increase of strength of *Tinctura Buchu* from 1:8 to 1:5, and further *Infusum Buchu*, which at that point was unique to the BP (Squire, 1899). Culbreth, in his 1906 edition of *Manual of Materia Medica and Pharmacology*, provided a lengthy monograph for *Barosma betulina*, including the mention of common adulterants; at that point *B. crenulata* was no longer compendial (Culbreth, 1906).

*Buchu* and *Fluidextractum Buchu* were dismissed from the eleventh decennial revision (USP XI, 1930). *Buchu* (raw material) entered the sixth edition of the NF (NF VI, 1936). Monographs for preparations also remained compendial in the NF after dismissal from the USP: *Elixir Buchu*; *Elixir Buchu Compositum*; *Elixir Buchu et Potassii Acetatis*; *Elixir Buchu*, *Juniperi et Potassii Acetatis*; *Fluidextractum Buchu*; *Fluidextractum Buchu Compositum*. *Buchu* and preparations made from it last appeared in the tenth edition of the NF (NF X, 1955), in force from 1955 until 1960. Monographs for *buchu* and preparations made from it were dismissed from the eleventh edition of the NF (NF XI, 1960).

By 1899, *buchu* was still not compendial in Austria, Germany, Hungary, Italy, Russia or Switzerland (Squire, 1899). Nonetheless, the inaugural edition of *Hager’s Handbuch der Pharmaceutischen Praxis* (1880), a monumental reference for the pharmacist, which has been in print and updated regularly to this day, contained a detailed monograph for *Barosma* (Hager, 1880) noting its reluctant use in Central Europe despite it purportedly being more efficacious than bearberry [*Arctostaphylos uva-ursi* (L.) Spreng]. According to Schneider (1974), *Folia Bucco* appeared in the supplements to the German pharmacopeia (DAB) around 1900. Madaus (1938), in another seminal compendium, the *Lehrbuch der biologischen Heilmittel*, provided a detailed account for *bucco*, including a summary of its etymology, botany, history, uses, and directions of use, based on US and UK compendial monographs. He mentioned its presence in the supplement to DAB VI, which, however, was only published in 1941. In fact, the first compendial monograph for Germany *Extractum Bucco fluidum* was published in the supplement to DAB IV (Anonymous, 1916).

The 1949 *British Pharmaceutical Codex* (BPC) listed buchu, specifically *Infusum Buchu Concentratum* and *Infusum Buchu Recens* as per BP 1932, and *Tinctura Buchu*. Noteworthy is the entry on action and uses: “[It] is now superseded by sulfonamides and penicillin […]” (Anonymous, 1949). It was still present in the BPC 1963 (Wade, 1977). While it is unclear exactly when it was omitted from the BP—it was no longer compendial by 1992—*buchu* retained its spot in the *British Herbal Pharmacopoeia* throughout all three editions (1971ff) (Scientific Committee, 1983). A 1997 query of the UK Medicines Control Agency’s (now Medicines and Healthcare Products Regulatory Agency) database of registered products yielded 17 products (liquid and solid dosage forms) of 10 manufacturers containing *buchu* as a sole ingredient or in combinations. All products had been licensed as GSL (General Sales List) between 1988 and 1996 (unpublished data). Only two products transitioned into the Traditional Herbal Medicinal Product category post 2004, however, neither are actively marketed. Nonetheless, the UK compendium *Herbal Medicines* (Barnes et al., 2013) includes a monograph for *buchu* to this day. Other European national pharmacopoeias also retained *buchu*, e.g., the *Pharmacopée Française* (ANSM, 1983) only earmarked it for omission in 2015, but keeps it currently listed in an addendum.

To inform the German *Kommission* *E* monograph (Kommission, 1990), an unpublished report was compiled (Franz and Hoth, 1987) in 1987. It contained a summary of botanical, biochemical and pharmacological data. It can be assumed that the decision of *Kommission E* to publish a “negative” monograph for *buchu:* “*Since the claimed effectiveness has not been documented, the application of buchu leaf cannot be recommended. The use of buchu leaf as an aroma or flavor corrigent in tea mixture is acceptable,*” was based on the absence of toxicological and clinical data. At the time, six combination products containing *buchu* leaf were on the German market. The *Kommission E* verdict effectively dealt a ‘death sentence’ to *buchu* products in Germany, as health claims could no longer be assigned to products and by 1994 medicinal products containing *buchu* had largely disappeared (Anonymous, 1994). The German drug database *AMIce* lists a total of 129 products containing *buchu* which are no longer licensed. The only remaining are homoeopathic products as per the first German homoeopathic pharmacopoeia (HAB 34) (Schwabe, 1934), albeit *buchu* having become obsolete and no longer listed in HAB 1 (Anonymous, 1978). It does, however, remain compendial in the *Homeopathic Pharmacopoeia of US* to this day.

## *Buchu* Today

The 1990s saw some revival of interest in the medicinal properties of *buchu*, but primarily in its country of origin, meanwhile it was still traded as a flavour in both Europe and the US (Simpson, 1998; Cohen et al., 2020). In 1999, the Agricultural Research Council (ARC) of South Africa initiated a project to cultivate *buchu* commercially to prevent it from becoming extinct (Coetzee, 1999). Demand for *buchu* oil increased significantly from the beginning of 1990, and with cultivation proving difficult, price hikes led to unsustainable harvesting practices. To remedy this situation, local manufacturers like Afriplex (Pty) Ltd. and Puris Natural Aroma Chemicals (Pty) Ltd. initiated numerous cultivation projects, e.g., by 2008, Afriplex had 82 ha with 3.5 million *buchu* plants under cultivation (Afriplex, 2008). In 2009, Bhat and Moskowitz identified eight commercial tea products containing *buchu* in the South African market (Bhat and Moskovitz, 2009). In 2011, Lubbe and Verpoorte noted the production volume for essential oil of *buchu* to be in the 1–50 tons per annum range, in 2010 the price for raw material was given at US$ 56/kg (Lubbe and Verpoorte, 2011). A few recent reviews point out the potential as an herbal medicinal product (Moolla and Viljoen, 2008; Street and Prinsloo, 2013; Skosana et al., 2014; Huisamen et al., 2019), however, from an international perspective the sleeping beauty is still waiting for its prince.

### Phytochemical Composition

Taxonomically, the two species *A. betulina* and *A. crenulata* can be mainly distinguished by their leaf form. In addition, the cultivation of both species outside their natural habitat resulted in the formation of hybrid plants (Collins et al., 1996). The phytochemical composition of the leaf oil of *A. betulina* and *A. crenulata* was extensively investigated by several research groups applying gas chromatography (GC) coupled to a flame ionisation detector or mass spectrometer including also cluster analysis. Fluck et al. identified pulegone and diosphenol as constituents of *buchu* oil (Fluck et al., 1961). Lamparsky and Schudel isolated 8-mercapto-*p*-menthan-3-one, a sulphur-containing terpene responsible for the flavour and aroma of the oil (Lamparsky and Schudel, 1971). Kaiser et al. identified more than 120 components including the already known pulegone, diosphenol and 8-mercapto-*p*-menthan-3-one (Kaiser et al., 1975). Collins et al. detected a total of 56 compounds including 14 new and found pulegone to be the key identification marker. They described two chemotypes for *A. betulina* depending on the diosphenol and isomenthone content. The diosphenol chemotype is characterized by high (ψ)-diosphenol (>10%) and diosphenol (>12%) and low isomenthone concentrations (<28%), the isomenthone chemotype by high isomenthone (>31%) and low (ψ)-diosphenol (<0.16%) and diosphenol (<0.14%). No chemotypes were found for *A. crenulata* oil (Collins et al., 1996). In their study of the chemical composition of *A. betulina*, *A. crenulata* and their hybrid, Posthumus et al. (1996) identified several rare bi- and tri-functionalized monoterpenes besides the commonly known monoterpenes. These included hydroxylated diosphenols, several hydroxymenthones and some acetates thereof. 8-hydroxy-4-menthen-3-one and 8-hydroxy-menthone were suspected to be decomposition products of pulegone (Posthumus et al., 1996). Finally, Viljoen et al. were able to confirm the data obtained from the previous phytochemical studies (Viljoen et al., 2006). Relevant findings are summarized in Table 3.

**TABLE 3:** Comparable overview of the phytochemical composition of the essential oil determined for *A. betulina* and *A. crenulata* and the hybrid of both.

Study	*A. betulina*	*A. crenulata*	Hybrid
Fluck et al. (1961)	Limonene, menthone, diosphenol, *l*-pulegone, (ψ)-diosphenol (an isomer of diosphenol)	Limonene, menthone, traces of diosphenol, *l*-pulegone, (ψ)-diosphenol	n.a
Kaiser et al. (1975)	Limonene 17%	Limonene 9%	n.a
	Menthone 17%	Menthone 6%	
	Isomenthone 43%	Isomenthone 22%	
	Isopulegone 4%	Isopulegone 10%	
	Pulegone 3%	Pulegone 50%	
	ψ-diosphenol 8%	ψ-diosphenol 1%	
	Diosphenol 9%	Diosphenol 1%	
	8-mercapto-*p*-menthan-3-one ++	8-mercapto-*p*-menthan-3-one +	
	8-acetylthio-*p*-menthan-3-one +	8-acetylthio-*p*-menthan-3-one ++	
Collins et al. (1996)	Pulegone 2.4–4.5% 8-mercapto-*p*-menthan-3-one > 8-acetylthio-*p*-menthan-3-one *cis*-8-mercapto-*p*-menthan-3-one > *trans*-8- mercapto-*p*-menthan-3-one	Pulegone 31.6%–73.2% 8-acetylthio-*p*-menthan-3-one > 8-mercapto-*p*-menthan-3-one	Simultaneous presence of relatively high concentrations of both pulegone and diosphenol 8-mercapto-*p*-menthan-3-one > 8-acetylthio-*p*-menthan-3-one *cis*-8- mercapto-*p*-menthan-3-one > *trans*-8- mercapto-*p*-menthan-3-one
Posthumus et al. (1996)	(Iso)menthone 31% ψ-diosphenol 41% *cis*- and *trans*-8- mercapto-*p*-menthan-3-one 3% + two decomposition products of pulegone 8-hydroxy-4-menthen-3-one 8-hydroxy-menthone	Pulegone 54% *trans*-acetylthio-*p*-menthan-3-one 7 + two decomposition products of pulegone 8-hydroxy-4-menthen-3-one 8-hydroxy-menthone	Intermediate composition including (iso)menthone 55%
Viljoen et al. (2006)	Limonene 23.7% Menthone 29.2% Isomenthone 14.2% Pulegone 8.4% Diosphenol 2.5% *cis*-8-mercapto-*p*-menthan-3-one 0.1 *trans*-8-mercapto-*p*-menthan-3-one 0.1%	Limonene 13.4% Menthone 16.6% Isomenthone 7.3% Diosphenol 0.1% *cis*-8-mercapto-*p*-menthan-3-one *trans*-8-mercapto-*p*-menthan-3-one 0.1%	n.a

n.a., not analyzed.

Whereas most of the monoterpenes identified in *buchu* oil may be also found in many other plants, *buchu* is the only genus that produces diosphenol, responsible for the distinctive blackcurrant flavor; hence its use in the food industry to enhance fruit flavors in sweets and beverages (Moolla and Viljoen, 2008).

The presence of diosphenol as a distinctive constituent of *A. betulina* essential oil in addition to pseudo-diosphenol, limonene, 1,8-cineole, menthone, isomenthone and *trans*-8-mercapto-*p*-menthan-3-one was also confirmed in a recent quality control protocol published as part of a monograph by Viljoen and colleagues. Chemical profiling of a methanol extract of *A. betulina* revealed the presence of hesperidin, rutin, and diosmin, all serving as non-volatile marker compounds (Viljoen et al., 2021).

### Pharmacological Activity

Pharmacological experiments in the early 2000s were mostly carried out with the hydro-distilled essential oils and/or methanol-dichloromethane (1:1) extracts of both *Agathosma* species.

#### Antimicrobial Activity

Based on the traditional use of *buchu* in urinary tract infections, several assays have been utilized to study the antimicrobial activity of the hydro-distilled essential oils and methanol-dichloromethane (1:1) extracts of *A. betulina* and *A. crenulata*. It is noteworthy, however, that these extracts do not correspond with traditional extraction methods as an infusion in water or a tincture in ethanol.

Applying the micro-titre plate dilution method in the minimum inhibitory concentration (MIC) assay, the methanol-dichloromethane (1:1) extracts of both species revealed moderate antimicrobial activity with a MIC in the range of 2 mg/ml–4 mg/ml against the tested pathogens *Bacillus cereus*, *Staphylococcus aureus*, *Klebsiella pneumoniae*, and *Candida albicans*. Much higher MIC values in the range of 3 mg/ml–32 mg/ml were determined for the essential oils of both species. Unfortunately these MIC values are too high and indicate low activity. By comparison the MIC of the respective controls ranged between 2.5 × 10^−3^ and 6.3 × 10^−3^ mg/ml (Viljoen et al., 2006; Moolla et al., 2007).

In another study by Lis-Balchin et al. utilizing the agar disc diffusion assay the essential oil of both species (10 µL undiluted) did not demonstrate antimicrobial activity against *Enterococcus hirae* and *Pseudomonas aeruginosa*, and very low activity against *Escherichia coli*, *Saccharomyces cerevisiae* and *S. aureus* (Lis-Balchin et al., 2001)*.* This is not surprising, since the selected assay is not suitable for essential oils, because of the lack of solubility in aqueous environments, which consequently results in low diffusion.

Steenkamp et al. investigated the antimicrobial activity of aqueous and ethanolic extracts of *A. betulina* on *E. coli* using the micro-well dilution method revealing no effect of either extract on the growth of *E. coli* (Steenkamp et al., 2006).

The methanol:dichlormethane extract (1:1) of *A. betulina* was re-investigated by Sandasi revealing MIC values ranging between 3 and 6 mg/ml against *Listeria monocytogenes, Pseudomonas aeruginosa*, *Candida albicans*, *Escherichia coli*, *Proteus vulgaris*, *Staphylococcus aureus*, and *Enterococcus faecalis* in the MIC microplate assay. It also prevented the growth and development of biofilms by preventing the attachment of bacteria to the polyvinyl chloride surface in the crystal violet (CV) assay, except for *C. albicans* (Sandasi, 2008).

Unfortunately, the phytochemical composition of the tested methanol-dichloromethane extract has not been investigated, so it can only be postulated that the antimicrobial effects observed may likely be attributed to inherent coumarins and flavonoids. But although antimicrobial acting flavonoids may be also expected to be present in the ethanolic extracts of *A. betulina* the latter revealed no effect. Obviously, the methanol-dichloromethane extract also contains other components that are not found in the aqueous and ethanolic extracts, which are responsible for the moderate antimicrobial activity. Also, the monoterpenes identified in the essential oils should have revealed more potent antimicrobial effects. The poor activity of the essential oils compared to the extracts was attributed by Moolla to the insolubility of the essential oil in the growth medium, a negative influence of the pH of the medium, or a possible inactivation of the essential oil components by constituents of the growth medium (Moolla, 2005).

For the time being it can thus be concluded that only the methanol-dichloromethane (1:1) extracts of both *Agathosma* species exert low to moderate activity against micro-organisms responsible for urinary tract infections like *E. coli* and *K. pneumoniae*. Aqueous or ethanolic extracts as well as the essential oils were shown to be inactive. The missing phytochemical characterization of the applied extracts prevents interrogating the causes for the observed differences.

#### Anti-Inflammatory Activity

In the 5-lipoxygenase (5-LO) assay the essential oils of *A. betulina* and *A. crenulata* revealed IC_50_ values of 50.37 ± 1.87 μg/ml and 59.15 ± 7.44 μg/ml, respectively, indicating low 5-LO inhibitory activity (Viljoen et al., 2006). Investigations on individual oil components that might contribute to the anti-inflammatory activity have not been carried out, but Moola and Viljoen assumed that the monoterpene limonene may be responsible for the observed inhibition of 5-LO *in vitro* as it is present in both species and is known for its anti-inflammatory effects (Moolla and Viljoen, 2008). It is worth mentioning that other *Agathosma* species like *A. collina* Ecklon & Zeyher and *A. namaquensis* Pillans revealed better 5-LO inhibitory activity, reflected in IC_50_ values corresponding to 25.98 ± 1.83 μg/ml and 31.54 μg/ml, respectively.

Aqueous and ethanolic extracts of *A. betulina* revealed a higher activity for the ethanolic extract (250 μg/ml) on cyclooxygenase (COX)-1 (98% inhibition) and COX-2 (25% inhibition). Both extracts were obtained by extracting 1 g of dried plant material with either 10 ml of water or ethanol in an ultrasound bath for 30 min, after which the extracts were filtered and evaporated to dryness (Steenkamp et al., 2006).

Other pharmacological targets playing a role in inflammation, e.g., microsomal prostaglandin E synthase-1 have not been investigated.

Apart from the limited *in vitro* investigations, one double-blind placebo-controlled study in 30 male participants reported reduction of swelling and pain in exercise-induced muscle damage following topical application of *A. betulina* oil containing gel three times a day (Lambert et al., 2002).

Taken together, the current data situation with regard to anti-inflammatory effects of the essential oil or aqueous and ethanolic extracts is very poor, thus more investigations including detailed phytochemical characterization of the tested oils or extracts are needed to come to a final conclusion with regard to the anti-inflammatory efficacy.

#### Analgesic Activity

Analgesic activity has been investigated by Chiguvare et al. in Swiss albino mice receiving 200 mg/kg of an ethanolic extract of *A. betulina* compared to 200 mg/kg silver nanoparticles prepared from the ethanolic extract, and 100 mg/kg of aspirin as positive control. The silver nanoparticles showed better analgesic properties than aspirin, reflected in a lower number of paw licks in the formalin test. The inhibition values for the silver nanoparticles synthesized at different temperatures ranged between 73% and 98% for the neurogenic phase and between 55% and 80% in the inflammatory phase. The inhibition values for the crude ethanolic extract were 55% and 45% for the neurogenic and inflammatory phase, respectively, compared to 84% and 81% for aspirin (Chiguvare et al., 2016).

Apart from a general screening on the presence of glycosides, flavonoids, alkaloids, terpenes, steroids, tannins, saponins and proteins in the ethanolic extracts no detailed phytochemical characterization of the individual components has been carried out. Thus, the significance of this single test for evaluating the analgesic activity of ethanolic *A. betulina* extracts is very limited.

#### Antioxidant Activity

The radical scavenging activity of methanol-dichloromethane (1:1) extracts of several *Agathosma* species has been tested in the 2,2-diphenyl-β-picrylhydrazyl (DPPH) and 2,2′-azino-di(3-ethylbenzthiazoline-6-sulfonic acid) (ABTS) assays. In the DPPH assay, both *A. betulina* and *A. crenulata* extracts showed poor antioxidant activity with IC_50_ values >100 μg/ml. In contrast, moderate activity with IC_50_ values corresponding to 37.75 ± 0.54 μg/ml and 33.32 ± 0.33 μg/ml was determined for both extracts in the ABTS assay. These seemingly contradictory results, however, point at the fact that mechanisms of both reagents are different and suggest interactions at different stages of the oxidative process (Moolla et al., 2007). The outcomes confirmed results of an earlier study (O’Brien, 2005), in which acetone, 80% methanol and aqueous extracts were tested for their antioxidant activities in the DPPH assay. The only extract that suppressed the oxidation of linoleic acid was the acetone extract containing the four lipophilic flavonoids 3 and 3,3′-dimethyl ethers of quercetin and the 3 and 3,4′ dimethyl ethers of kaempferol. The 80% methanol extract contained hyperoside, rutin and a novel compound designated agathosin, which was shown to be a quercetin glucoside esterified with oleuropeic acid. The main antioxidative compound in the aqueous extract was rutin. But despite the presence of quercetin, kaempferol and rutin, all of which have known radical scavenging properties, only a poor correlation could be found between the total phenolic content of the different extracts and the radical scavenging activity.

On the other hand, Steenkamp et al. determined a scavenging activity for hydroxyl radicals generated by a Fenton-type reaction for very high concentrations of the aqueous and ethanolic extract of *A. betulina* (4 mg/ml) corresponding to 80% using electron spin resonance spectrophotometry (Steenkamp et al., 2006). However, these concentrations are far away from being of relevance for practical application.

Thring et al. reported a TEAC of 11.8 µM Trolox when applying 25 µg aliquots of an aqueous extract of *A. betulina* (Berg) Pill. (10 mg/ml). The aqueous extract was obtained by extracting 500 mg dried ground herb in 10 ml boiling water, followed by sonication for 15 min, filtration on the following day, and drying with a fan. Trolox refers to the standard applied in the ABTS+ diammonium salt free radical assay. Here the correlation analysis showed a significant correlation (*p* = 0.001) between total phenolic content (0.246 mg/ml, as equivalents of gallic acid determined by the Folin-Ciocalteu assay) and TEAC. In the superoxide dismutase (SOD) assay *buchu* aqueous extract inhibition activity was reported at 20.49% compared to 85.02% of the positive control (SOD at 3.33 units final volume). SOD is a naturally occurring enzyme that protects the cell from the reactive and damaging superoxide anion (O_2_^−^) by dismuting it into O_2_ and H_2_O_2_ (Thring et al., 2009).

All experiments indicate that different *buchu* extracts may exert antioxidant effects in different experimental settings. Unfortunately, they were carried out without detailed analysis of the respective extract compositions, and applying too high concentrations, so no conclusions can be drawn regarding effective extract components and effective concentrations in practical applications. Further research is therefore needed.

#### Effects of Buchu Water on Metabolic Syndrome

A recent set of investigations carried out on the quest of Cape Kingdom Nutraceuticals addressed the efficacy of aqueous *buchu* extract (presumably from *A. betulina* and *A. crenulata*) on diseases related to metabolic syndrome in *in vitro* and *in vivo* test systems. The extract, marketed commercially as *buchu* water, is a water condensate recovered during the steam distillation of commercial *buchu* oil, back-extracted using ethyl alcohol followed by drying under reduced pressure and re-suspension in methanol. As the oil is distilled at low temperatures under vacuum, the water contains not only the water-soluble hydrophilic compounds but also some of the more volatile oil-soluble compounds. Targeted analysis using LC-MS revealed the presence of hesperidin, rutin, diosmin, quercetin and pulegone besides other molecular species with unknown structures (Huisamen et al., 2019). Unfortunately, no quantitative analysis was conducted.

##### In vitro Effects on Glucose Metabolism

The effect of the aqueous extract in comparison to pure *buchu* oil on excessive glucose utilization was tested on Chang Liver cell line of hepatocyte origin and 3T3-L1 cell line of adipose tissue origin. In the Chang Liver cell line only *buchu* oil showed a dose-dependent increase in the uptake of the additional glucose (1 mg/ml) added to the medium by 12–21% in the concentration range from 0.15–0.6 ppm. In comparison, the positive control metformin at 1 µM exhibited 23%–25% increase in uptake. In the 3T3-L1 cell line only the aqueous extract caused a 35%–40% increase in the glucose uptake, comparable to 35% observed increase obtained with 1 µM insulin serving as positive control. The observed difference in sensitivity of the tested cell lines to oil or aqueous extract is not surprising, as they also respond differently to well-established anti-glycaemic drugs like metformin or insulin depending on their origin (Huisamen et al., 2019).

##### Anti-Inflammatory Activity

In human peripheral blood cells, the extract caused a significant inhibition of the respiratory burst of neutrophils and monocytes and of the expression of adhesion molecules (CD11b/CD18) in the range of 1:400 to 1:3,200 dilution of a 600 μg/ml stock solution. The latter effect was more pronounced in neutrophils than in monocytes. Furthermore, the extract inhibited the release of potent cytokines like interleukin (IL)-6 and tumour necrosis factor (TNF)-α. In general the round leafed *buchu* extract was more effective than the oval leafed *buchu* extract in three of the total extract fractions tested, only in one fraction the oval leafed *buchu* extract showed a greater inhibitory activity (Huisamen et al., 2019). However, these results are very difficult to comprehend since detailed information on the preparation and phytochemical composition of the different fractions of the total aqueous extract are missing. Moreover, these results have not been verified by any other research group.

Surprisingly, the above results were considered sufficient to verify the observations made *in vitro* in animal models, although no mechanism of action can be deduced from the *in vitro* experiments and no identification of the effective compound(s) was carried out.

##### Anti-Diabetic, Anti-Obesity, Anti-Hypertensive Effects of Buchu Water in Animals

To investigate anti-diabetic effects, type 1 diabetes (T1D) was induced in adult male Wistar rats by injecting streptozotocin leading to the chemical destruction of 50% the pancreatic β-cells. Free access to diluted *buchu* water was given 3 weeks after the streptozotocin injection for a duration of 14 weeks. For inducing type 2 diabetes (T2D) the diet-induced obesity (DIO) model was applied in young rats by administering rat chow diet supplemented with sugar and condensed milk for 16 weeks. *Buchu* water treatment started 8 weeks after receiving the DIO diet and continued for the remaining 8 weeks.

In T1D rats with glucose levels <20 mmol/L the aqueous *buchu* extract completely normalized glucose levels and in T1D rats with glucose levels >20 mmol/L, they were significantly reduced compared to the untreated T1D group. The T2D rats ingesting *buchu* water did not accumulate additional intraperitoneal fat as happened in the DIO group not receiving *buchu* water. The whole-body glucose tolerance revealed no significant differences between control animals and DIO animals that ingested *buchu* water. The insulin sensitivity at organ level measured in isolated ventricular cardiomyocytes was significantly enhanced in the control and DIO group receiving *buchu* water. The insulin secretion was enhanced in the DIO group receiving *buchu* water, accompanied by a significant increase in the C-peptide levels and a significant upregulation of the pancreatic transcription factors musculoaponeurotic fibrosarcoma homolog A (Maf A) and pancreatic duodenal homeobox 1 (Pdx-1). The latter is an indication for the regeneration of pancreatic β-cells.

Since both animal models represent so-called pre-stages of type 1 and type 2 diabetes, it was concluded that *buchu* water can be used as treatment option in newly diagnosed pre-type 1 diabetic patients. It must, however, be emphasized that human T1D, in contrast to the chemically induced TD1 in rats, is an autoimmune disease where the regeneration of pancreatic β-cells may be counteracted by autoantibodies. Because of the generally known low bioavailability of the very low concentrated polyphenols in the ingested aqueous *buchu* extract, it was hypothesized that *buchu* extract may exert influences on the gut microbiome, which in turn could produce substances like γ-aminobutyric acid (GABA), known to act on the transcription factors involved in pancreatic redifferentiation (Huisamen et al., 2019). Nonetheless, much more work is required to verify this hypothesis.

Based on the observed effects of aqueous *buchu* extract on intraperitoneal fat, the anti-obesity effect was studied in rats receiving a high-fat diet (HFD) for 16 weeks that was composed of normal rat chow supplemented with 10% saturated fat, 10% fructose, 10% casein and 1% cholesterol, rendering animals insulin-resistant and hypertensive. The HFD significantly increased the rats’ body weight from 381.3 ± 9.5 g (control group) to 451.8 ± 15.1 g (HFD group) and the intraperitoneal fat from 8.6 ± 0.4 g (control group) to 24.5 ± 0.7 g (HFD group). The consumption of *buchu* water resulted in less weight gain corresponding to 394.1 ± 14.0 g and less intraperitoneal fat gain corresponding to 15.9 ± 1.3 g in the HFD group. The consumption of *buchu* water resulted in a significant reduction in fat cell size (*p* < 0.0001), as well as significantly lower leptin levels (*p* = 0.0003) compared to the HFD group without intake of *buchu* water. No significant differences were observed in the adiponectin, TNF-α and IL-6 levels. *Buchu* water specifically lowered the elevated mRNA levels of peroxisome proliferator-activated γ receptor (PPARγ) to control values but not that of PPARα in the HFD group. The total cholesterol levels in both the control and HFD group (*p* = 0.03) and the plasma triglycerides in the HFD group were reduced (*p* = 0.03).

Again, ingredients responsible for the observed effects could not be identified since the *buchu* water intake corresponded to a mean of 30 ml per day and the concentrations of the included flavonoids were very low at 0.0005 mg/L diosmin, 0.007 mg/L quercetin, 0.001 mg/L hesperidin, and 0.0035 mg/L rutin (Huisamen et al., 2019).

When HFD rats ingested *buchu* water, the initial rise in blood pressure declined to control values at week 14 and infarct development induced in the isolated perfused hearts by regional ischemia was significantly smaller compared to the HFD group with no *buchu* ingestion. Serum aldosterone levels, as an indicator of the renin-angiotensin-aldosterone system, could be significantly lowered to 259.7 ± 65.5 pg/ml compared to 619.9 ± 136.1 pg/ml in the HFD group without *buchu* ingestion (Huisamen et al., 2019).

These findings suggest positive effects of *buchu* water on glucose homeostasis, weight gain, intraperitoneal fat gain, blood pressure and cardio-protection. It should be noted that dosing in the animal studies was equivalent to a human weighing approximately 70 kg drinking 250 ml/day of the extract. However, in the absence of clinical trials, no findings exist that verify these observations that were made in rats in humans. Clinical studies are urgently needed, especially in view of the different pancreatic biochemistry and the difficulties in extrapolating the data obtained from rats to humans. Unless these clinical trials are conducted, the reported positive anti-diabetic, anti-obesity, and anti-hypertensive effects of buchu water in rats remain questionable in humans. Even in rats the described effects remain inconclusive because of the missing information on extract composition. They also remain questionable as long they are not further verified by independent research studies supporting these observations.

### Toxicity

The toxicity of methanol-dichloromethane (1:1) extracts of *Agathosma* species was evaluated by Moolla et al. using the MTT [3-(4,5-dimethyl-2-thiazol-yl)-2,5-diphenyl-2H-tetrazolium bromide] cellular viability assay. In this assay several dilutions of methanol-dichloromethane (1:1) extracts displayed different degrees of cellular inhibition, but extracts of *A. betulina* and *A. crenulata* were not toxic at concentrations up to 100 μg/ml (Moolla et al., 2007).

The essential oils of both species proved to exhibit higher toxicity at the concentration tested, both having IC_50_ values of <0.0001 μg/ml (Viljoen et al., 2006). Essential oils at high doses were found to be hepatotoxic in rats, affecting liver and uterine functions. This was attributed to *R*-(+)-pulegone which is known to be a hepatotoxic compound causing depletion of glutathione at high doses. This depletion along with excess pulegone leads to centrilobular hepatocellular necrosis (Moolla, 2005). Caution should be exercised with essential oil from *A. crenulata* that contains higher amounts of pulegone. Pulegone is not water soluble, thus this caution does not apply to water extracts. Pulegone has been approved by the US FDA for use in the food industry (with a FEMA GRAS status) and is listed among the authorized synthetic flavouring substances (CFR 21–172.515). The no effect level of pulegone in beverages as stated in the *Opinion of the Scientific Committee on Food on the risks to human health of Polycyclic Aromatic Hydrocarbons in Food* is 100 mg/kg (European Commission, 2002). Consequently, the aqueous *buchu* extract applied in above animal experiments containing 74.22 mg/L ± 1.1 mg/L pulegone is well below the acceptable level indicated by the FDA and the LC_50_ value of 25.91 mg/ml determined by Raza et al. (2016).

### Market Presence of *Buchu* Products

As mentioned before, *buchu* is currently primarily used in the fragrance and flavour industry due to its sulphur-containing compounds and sensory properties.

Despite preliminary research suggesting pharmacological potential regarding its antimicrobial, anti-inflammatory, and antioxidant properties, *buchu* has not retained its place in the mainstream market. This is unsurprising as many of the effects have only been observed at exorbitantly high doses in the respective animal models. Effects reported for aqueous *buchu* extracts from animal studies cannot be related to putative active ingredients either, as doses were too low for explaining any direct actions on pharmacological targets. At the same time, urgently needed clinical trials have not been conducted.

Nevertheless, *buchu* enjoys a reputation as a general health tonic and is promoted to possess anti-inflammatory, antioxidant and antibacterial properties. It is marketed as a dietary supplement in various forms including teas, dried, whole, and powdered leaves, liquid and powder extracts, oils, tinctures, waters, capsules, tablets, gels and creams, that are moreover often adulterated (Raman et al., 2015). Online *buchu* features among ten plants commonly claiming benefit in kidney diseases (Vamenta-Morris et al., 2014). It is found in herbal preparations sold OTC or online associated with following claims: “blood pressure support,” “support cardiovascular health,” “digestive support” and in combination with cranberry for “traditional urinary support,” “supports body’s health against bacteria in the urinary tract” as well as in combination with other herbal ingredients to “support kidney health” and “support urinary bladder health.” This is alarming because such claims have neither been widely investigated nor substantiated by peer review (Vamenta-Morris et al., 2014). At the same time scientific literature has to date failed to prove any benefit in humans.

Other than in medicinal applications, *buchu* has recently found use in the green synthesis of nanoparticles (Thema et al., 2015; Pal et al., 2019). This opens up novel dimensions in the field of biotechnology and nanomedicine. Whereas physical and chemical routes are associated with high energy consumption, low yield, high cost, and environmental damage, biological pathways using plants or plant-based extracts as chelating agents represent a cost effective, simpler and eco-friendly approach. In fact the biosynthesis approach has been demonstrated to be effective in the synthesis of metal and oxide nanoparticles as e.g., the green synthesis of cadmium oxide nanoparticles based on *Hibiscus subdariffa* flower extract (Thovhogi et al., 2016), the green synthesis of silver nanoparticles of the crude extract of *Syzgium aromaticum* (Venugopal et al., 2017a), the green synthesis of SnO_2_ nanoparticles via *Aspalathus linearis* (Diallo et al., 2016), the green synthesis of samarium oxide nanoparticles via *Callistemon viminalis* extract (Sone et al., 2015), the biosynthesis of *Beta vulgaris* extract mediated silver nanoparticles with enhanced anticancer activity (Venugopal et al., 2017b), and the synthesis of single-phase α-Cr_2_O_3_ nanoparticles using *Callistemon viminalis*’ red flower extract (Sone et al., 2016), just to mention a view.

This is of great importance as it opens up new advantageous applications in biomedical, drug delivery, and food industries as well as in agriculture, and textile industries.

## Summary and Discussion

The popularity of *buchu,* i.e., *A. betulina* and *A. crenulata* in medicinal use may stem from its traditional use by the indigenous peoples of South Africa. This medicinal application was successfully disseminated by settlers, colonists, and explorers, which led to the tremendous popularity in Europe and the United States in the 19th century. However, because of the sparsity of scientific evidence and the advent of antibiotics the interest in buchu began to wane in the 20th century.

Whereas the phytochemical differences between the two *Agathosma* species have been well illustrated, recent pharmacological studies unfortunately could not alleviate the justified doubts regarding the traditional use of buchu for the treatment of urinary tract infection. Hence the overall modest number of assays addressing the antimicrobial activity revealed only low to moderate effects against micro-organisms responsible for urinary tract infection for a methanol-dichloromethane (1:1) extract that was not used traditionally and no effects for the essential oil or aqueous or ethanolic extracts. Taken together, the results of the antimicrobial tests remain inconclusive because the tentative active coumarins and flavonoids inherent in the different extracts have not been identified, preventing thus a direct comparison. Surprisingly also the monoterpenes in the essential oils failed to exert any antimicrobial effects they are known for. This was explained by the insolubility or reciprocal inactivation of the inherent ingredients in buchu by the ingredients in the growth medium. However, no further investigations were carried out to verify or deny these hypotheses.

ther pharmacological assays devoted to the study of anti-inflammatory, analgesic and antioxidant effects must be considered to be of limited scientific value, which is attributed to the very small number of potential pharmacological targets that have been tested and the extremely high doses applied being far away from doses applied in daily practice.

Recent studies on the effect of aqueous *buchu* extract on the metabolic syndrome should also be treated with caution as the promising results observed in animal studies may not be readily extrapolated to humans because of different pathophysiologies and the general absence of clinical trials.

Therefore, to answer the question whether *buchu* has been rightfully forgotten or has been just underutilized more studies of high scientific value are needed. Thus, extracts of *buchu* should be subjected to well-designed experiments *in vitro* and *in vivo* taking into consideration at first instance a detailed phytochemical characterization of the extract components and the application of clinically relevant doses. Based on the above future research activities should focus on the methanol-dichlormethane (1:1) extract of *A. betulina*, as it showed the most promising results in the antimicrobial studies carried out. In case of verified positive effects *in vitro*, the results obtained should be further tested for efficacy in urinary tract infections in randomized, double-blind, and placebo-controlled clinical trials. Also various promising antioxidant pharmacological activities and health benefits reported for citrus flavonoids (Mahmoud et al., 2019) like hesperidin (Li and Schluesener, 2017) and diosmin (Zheng et al., 2020) should be considered for *A. betulina*, as it also contains these components. In this context the effects of *A. betulina* on lowering the risks for degenerative diseases like cancer, cardiovascular diseases, Alzheimer’s disease and Parkinson disease should be further investigated. In case of verified pharmacological activity *in vitro* and in human *A. betulina* extracts may be utilized as food supplements for its preventive effects on degenerative diseases. In general, *A. betulina* should be preferred over *A. crenulata*, because of the lower amounts of the hepatotoxic pulegone, inherent to both species. Moreover also other species like *A. collina* or *A. namaquensis* should receive an increasing focus of attention. Results obtained should be verified in randomized, double-blind, and placebo-controlled clinical trials. But until these studies are available the answer to the question “rightfully forgotten or underutilized” remains open.

## Conclusion

Taken together, pharmacological studies carried out to date failed to confirm the traditional use or historical popularity of *buchu*. Hence, products containing *buchu* should be treated with caution in that the deduced effects of *buchu* may not occur. Only on the basis of well characterized extracts and realistic doses applied in pharmacological assays and clinical trials a final verdict on the therapeutic potential of *buchu* can be made, resulting in an appropriate classification as a dietary supplement or medicinal product with proven pharmacological activity. In this context future studies should focus on the antimicrobial and antioxidant effects of *A. betulina* extracts providing the base for more evident use of buchu in the treatment of urinary tract infections or as food supplement in degenerative diseases. Also the application of *A. betulina* in the green synthesis of nanoparticles should receive more attention in the future.

## Footnotes

1
http://lloydbleekcollection.cs.uct.ac.za/.
